# Dihydromyricetin Attenuates Depressive-like Behaviors in Mice by Inhibiting the AGE-RAGE Signaling Pathway

**DOI:** 10.3390/cells11233730

**Published:** 2022-11-22

**Authors:** Jun Huang, Bin Chen, Hao Wang, Sheng Hu, Xudong Yu, James Reilly, Zhiming He, Yong You, Xinhua Shu

**Affiliations:** 1 School of Basic Medical Sciences, Shaoyang University, Shaoyang 422000, China; 2 Department of Neurology, The Second Affiliated Hospital of Hainan Medical University, Haikou 570100, China; 3 The First Affiliated Hospital, Hengyang Medical School, University of South China, Hengyang 421001, China; 4 Department of Biological and Biomedical Sciences, Glasgow Caledonian University, Glasgow G4 0BA, UK

**Keywords:** depression, dihydromyricetin, inflammation, network pharmacology, AGE-RAGE signaling pathway

## Abstract

Depression is a complex mental disorder, affecting approximately 280 million individuals globally. The pathobiology of depression is not fully understood, and the development of new treatments is urgently needed. Dihydromyricetin (DHM) is a natural flavanone, mainly distributed in *Ampelopsis grossedentata.* DHM has demonstrated a protective role against cardiovascular disease, diabetes, liver disease, cancer, kidney injury and neurodegenerative disorders. In the present study, we examined the protective effect of DHM against depression in a chronic depression mouse model induced by corticosterone (CORT). Animals exposed to CORT displayed depressive-like behaviors; DHM treatment reversed these behaviors. Network pharmacology analyses showed that DHM’s function against depression involved a wide range of targets and signaling pathways, among which the inflammation-linked targets and signaling pathways were critical. Western blotting showed that CORT-treated animals had significantly increased levels of the advanced glycation end product (AGE) and receptor of AGE (RAGE) in the hippocampus, implicating activation of the AGE-RAGE signaling pathway. Furthermore, enzyme-linked immunosorbent assay (ELISA) detected a marked increase in the production of proinflammatory cytokines, interleukin-1 beta (IL-1β), IL-6 and tumor necrosis factor-alpha (TNFα) in the hippocampus of CORT-treated mice. DHM administration significantly counteracted these CORT-induced changes. These findings suggest that protection against depression by DHM is mediated by suppression of neuroinflammation, predominantly via the AGE-RAGE signaling pathway.

## 1. Introduction

Depression is a common and severe psychiatric disease with a prevalence of 5% in adults, affecting more than 350 million individuals across the world [1]. It is a complex mental disorder, associated with psychological, environmental and genetic factors [2]. Although there have been a great many studies, the underlying molecular mechanisms of depression formation and progression are poorly understood. Currently, a variety of antidepressants are available, e.g., tricyclics and monoamines, which have slow onset, low efficacy and undesirable side effects. Furthermore, less than two-thirds of patients respond to pharmacotherapy, while 70% of treated patients did not achieve full remission [3]. Therefore, there is an urgent need to develop more natural antidepressants with reliable efficacy and fewer side effects.

Dihydromyricetin (DHM) is a flavonoid produced from *Ampelopsis grossedentata (A. grossedentata)*, which is also present in other medicinal plants, e.g., *Cedrus deodara* and *Hovenia dulcis*, and in some plant-based foods, such as grapes [4]. DHM has demonstrated anti-inflammatory, anti-oxidative damage, anti-alcoholic and anti-cancer effects, and alleviates pathology in diabetes, skin damage, liver, kidney and cardiac injury [4,5]. Additionally, DHM has also shown neuroprotection in neurodegenerative disorders, e.g., Alzheimer’s disease and Parkinson’s disease [5,6]. Recently, DHM has been shown to have anti-depressive properties in rodents with diabetic and depressive symptoms [7]. However, the antidepressant-like effects of DHM have not been fully elucidated, especially in the chronic corticosterone-induced depression model.

Corticosterone is a rodent stress hormone, which is homologous to human cortisol. Corticosterone mediates the hypothalamic–pituitary–adrenal (HPA) axis and regulates brain functions in rodents [8]. Chronic corticosterone injection causes dysfunction of the HPA axis and induces depressive-like behaviors, including social withdrawal, anhedonia and despair, as well as increased pro-inflammatory responses and upregulated pro-inflammatory cytokines in rodents [3,8,9]. Chronic administration of corticosterone in rodents causes impaired circadian rhythms, increased oxidative damage and inflammation, decreased production of neurotrophic factors (e.g., brain-derived neurotrophic factor, BDNF) and reduced hippocampal neurogenesis, which are supposed to contribute to depression [3]. This chronic corticosterone exposure-induced depressive model has the advantages of simple replication and strong stability, and avoids the potential habituation effect of rodents to stress. This model has been widely used to examine the antidepressant-like role of drugs in rodents [3]. In the current study, we used the corticosterone-induced depressive mouse model to examine the effects of DHM on depression-like behavior and to investigate the underlying pathological mechanisms.

## 2. Material and Methods

### 2.1. Animals

Seven-week-old male Institute of Cancer Research (ICR) mice (30 ± 2 g weight) were purchased from Hunan SJA Laboratory Animal Co. Ltd. (Hunan, China) and pair-housed in a standard environment with sufficient water and food provision. Prior to the commencement of the experiments, the mice were petted for 5 min per day for a week by the experimenter. All animal treatment procedures were performed following the Guidance for the Care and Use of Laboratory Animals, University of South China.

### 2.2. Chemicals

Dihydromyricetin (Shanghai Yuanye Bio-Technology Co., Ltd., Shanghai, China) was dissolved in 0.9% saline containing 0.2% dimethylsulfoxide (DMSO). Corticosterone (Shanghai Aladdin Biochemical Technology Co., Ltd., Shanghai, China) was dissolved in 0.9% saline containing 0.1% DMSO and 1% Tween 80. Both dihydromyricetin (intraperitoneally, i.p.) and corticosterone (subcutaneously, s.c.) were selected at a dose of 20 mg/kg body weight in current experiments.

### 2.3. Animal Treatment

**Figure 1:**
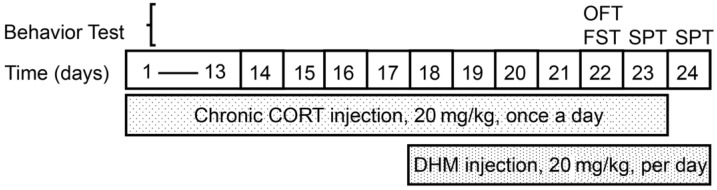
Schematic of the experimental design showing the timeline of drug injection and behavior tasks. CORT, corticosterone; DHM, dihydromyricetin; OFT, open field test; FST, forced swimming test; SPT, sucrose preference test.

The timeline of drug treatment is shown in Figure 1. Animals were randomly divided into three groups: control group (Control, n = 10), corticosterone group (CORT, n = 10) and corticosterone + dihydromyricetin group (CORT+DHM, n = 10). Animals in the control group received an injection (s.c.) of 0.9% saline containing 0.1% DMSO and 1% Tween 80 for 23 consecutive days (day 1–day 23) and an additional injection (i.p.) of 0.9% saline containing 0.2% DMSO for 7 consecutive days (day 18–day 24); The animals in the CORT group received 23 consecutive s.c. injections of CORT (20 mg/kg body weight, day1–day 23); animals in the CORT + DHM group received 23 consecutive s.c. injections of CORT (day 1–day 23) and 7 consecutive i.p. injections of DHM (20 mg/kg body weight, day 18–day 24). For behavior experiments, an open field test (OFT) and forced swimming test (FST) were conducted from 10:00 to 14:00 on day 22, while a sucrose preference test (SPT) was carried out at all times on days 23 and 24.

### 2.4. Behavior Tasks

#### 2.4.1. Open Field Test (OFT)

For the OFT, mice were randomly placed into open field apparatus (50 × 50 × 70 cm) for 5 min. The total distance traveled was measured using behavioral analysis software (Anymaze 6.16).

#### 2.4.2. Forced Swimming Test (FST)

For the FST, mice were individually placed into a plexiglass cylinder (25 cm high, 15 cm in diameter) containing 17 cm deep water (27 ± 1 °C) for 6 min. The behavior of these animals was video-recorded, and the immobility time was analyzed.

#### 2.4.3. Sucrose Preference Test (SPT)

The SPT was performed according to a previous description [9]. Briefly, the SPT was divided into two phases (habituation and test phases). During the habituation phase, the mice were exposed to two identical bottles containing 1% sucrose solution for 24 h. The next 24 h was the test phase, during which mice were presented with two identical bottles, one containing 1% sucrose solution and the other containing only water. Sucrose preference was designated as the volume of sucrose intake/(sucrose intake + water intake) × 100% during the test phase.

### 2.5. Western Blotting

To avoid the possible influence of the behavioral tests on expression of target proteins, another cohort of animals was set up and received same treatment as described in Section 2.3 without the behavior task. These animals were killed 1 h after the final DHM treatment on day 24. The entire hippocampus tissues were carefully dissected and lysed in the RIPA buffer with protease inhibitors and phosphatase inhibitors. The concentration of lysed samples was measured using a commercial kit following the manufacturer’s protocol. An amount of 50 µg protein of individual samples was separated using sodium dodecyl-sulfate polyacrylamide gel electrophoresis (SDS-PAGE) and transferred onto PVDF (polyvinylidene difluoride) membranes. After blocking with 3% BSA at room temperature for 1 h, the membranes were exposed to anti-AGE, anti-RAGE (Abcam, Cambridge, UK, 1:1000) or anti-β-actin (Proteintech, Rosemont, IL, USA, 1:5000) antibodies at room temperature for 2 h, followed by exposure to secondary antibodies for 2 h. The signals were detected using the ChemiDoc XRS imaging system (Bio-Rad, Hercules, CA, USA) and the intensity of targeted bands was quantified and normalized to β-actin.

### 2.6. Enzyme-Linked Immunosorbent Assay (ELISA)

IL-1β (Cat. KE10003), IL-6 (Cat. KE10007) and TNFα (Cat. KE10002) ELISA kits were purchased from Proteintech (https://www.ptglab.com/, accessed on 5 July 2022). The procedure was performed according to the manufacturer’s guidance. Briefly, 100mg mouse hippocampus tissue from individual animals was homogenized in 1 mL extraction reagent containing 1 mM phenylmethylsulfonyl fluoride and centrifuged at 10,000× *g* for 10 min at 4 °C. The supernatants were collected, and the concentration was determined using a commercial kit. An amount of 100 µL of standards or samples was added to the wells of capture antibody-coated microplate and incubated for 1 h at 37 °C. The liquid was removed, and the plate was washed four times with 1× washing buffer. An amount of 100 µL 1× detection antibody was added to each well and incubated for 1hr at 37 °C. The excess antibodies were washed away, and the plate was washed four times with 1× washing buffer. An amount of 100 µL of 1× horseradish peroxidase (HRP)-conjugated antibody was added to each well and was incubated for 40 min at 37°C. Excess antibody was washed away with 1× washing buffer. An amount of 100 µL of 3,3’,5,5’-Tetramethylbenzidine (substrate for HRP) was added to each well and incubated in darkness for 15 min at 37 °C. Additionally, 100 µL of stop solution was added to each well and the signal was determined using a microplate reader.

### 2.7. Network Pharmacology Analyses

#### 2.7.1. Prediction of DHM and Depression Targets

We used the PubChem database (https://pubchem.ncbi.nlm.nih.gov/, accessed on 15 August 2022) to search the structures of canonical SMILES strings of DHM, which were imported into the Swiss Target Prediction platform (http://www.swisstargetpredict.ch/index.php, accessed on 15 August 2022) and TargetNet database (http://targetnet.scbdd.com/calcnet/index/, accessed on 15 August 2022) to foreshadow their molecular targets.

The keyword “depression” was used for entering into the Genecards database (https://www.genecards.org/, accessed on 18 August 2022) (relevance score >1 was selected), TTD database (https://db.idrblab.org/ttd/, accessed on 18 August 2022) and OMIM database (https://omim.org/, accessed on 18 August 2022) and depression-related genes (species “Homo sapiens”) were screened.

#### 2.7.2. Construction of Venn Diagram and DHM Targets Depression Network

The common targets between DHM and depression were determined and visualized using the online tool Venny 2.1 (https://bioinfogp.cnb.csic.es/tools/venny/, accessed on 18 August 2022). Subsequently, the common targets were imported into Cytoscape software (version 3.7.2, https://cytoscape.org/, accessed on 18 August 2022) to construct a DHM targets depression network.

#### 2.7.3. Construction of Protein–Protein Interaction (PPI) Network

The 75 intersection targets between DHM and depression were input to the STRING database (https://string-db.org/ver.11.0, accessed on 18 August 2022) to identify the PPI relationships. In the current study, the data analysis mode was set as “multiple proteins”, the species were selected as “Homo Sapiens”, the combined scores ≥ 0.4 and the disconnected nodes were hidden. The network graphics were downloaded, and the analyzed results were saved as TSV format files and imported into Cytoscape 3.7.2 software for the network construction.

#### 2.7.4. Gene Ontology (GO) Function and Kyoto Encyclopedia of Genes and Genomes (KEGG) Pathway Enrichment

The intersecting targets were also imported into the R Studio software (https://www.rstudio.com/, accessed on 22 August 2022) and the “clusterProfiler” R package (https://bioconductor.org/packages/release/bioc/html/clusterProfiler.html, accessed on 22 August 2022) to annotate the gene ontology (GO) enrichment and KEGG pathways. The related biological processes and signaling pathways were identified via GO enrichment and KEGG pathway enrichment analyses. The *p*-value < 0.05 was set as the screening threshold of enrichment molecular analysis. The top ten GO terms in biological process, function, and cellular component and top twenty KEGG pathways were imported into the R Studio software (https://www.rstudio.com/, accessed on 22 August 2022) and visualized.

### 2.8. Statistical Analysis

All the data in the present experiment are expressed as mean ±SEM and analyzed by Sigma Plot 12.5 software using a one-way ANOVA (analysis of variance) test, followed by a post hoc Fisher LSD test. Data were regarded as significant if the *p*-value < 0.05.

## 3. Results

### 3.1. Dihydromyricetin Alleviated CORT-Induced Depressive-like Behaviors in Mice

**Figure 2:**
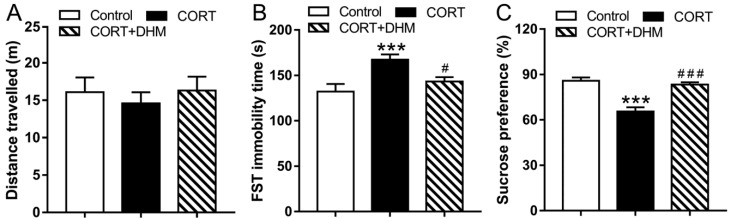
Effect of DHM administration on depression-like behaviors induced by CORT in mice. (**A**) Total distance travelled by the three groups in the open field test (OFT) (n = 10). (**B**) Immobility time of the three groups in the forced swimming test (FST) (n = 10). (**C**) Sucrose preference of the three groups in the sucrose preference test (SPT) (n = 5). Data are expressed as mean ± SEM; *** *p* < 0.001, vs. the control group; ^#^ *p* < 0.05, ^###^ *p* < 0.001, vs. the CORT group. CORT, corticosterone; DHM, dihydromyricetin.

We first investigated the effect of DHM on depressive-like behavior in animals receiving chronic injections. As shown in Figure 2, animals in the control group, the CORT group, or the CORT+DHM group had no significant differences in distance traveled in the open field test (OFT) (*p* > 0.05, Figure 2A). In the FST, the CORT group showed significantly increased immobility time compared with the control group, while DHM-treated animals had significantly less immobility time (*p* < 0.001, Figure 2B) compared to the CORT group; in the SPT, the animals in the CORT group had a significant decrease in sucrose preference, compared to the control group (*p* < 0.001, Figure 2C). Animals in the CORT + DHM group showed significantly increased sucrose preference (*p* < 0.001, Figure 2C) compared to the CORT group.

### 3.2. Target Prediction of DHM in Depression

**Figure 3:**
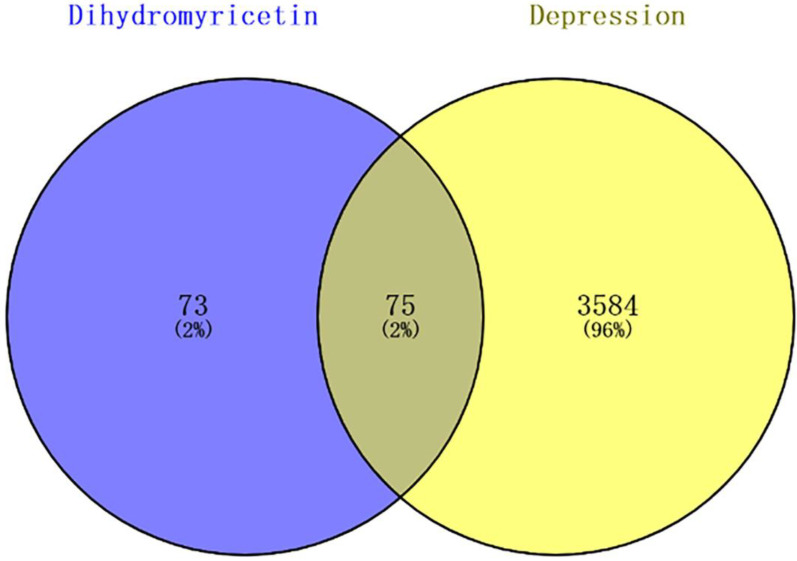
The intersection of Dihydromyricetin targets and depression targets.

To elucidate the underlying mechanisms of DHM against depression, a network pharmacology approach was applied. A total of 72 genes from the Swiss Target Prediction platform and 114 genes from the TargetNet database were identified as DHM targets. After removing duplicate genes, the final number of targeted genes was 148 (Table S1). A total of 3231 genes from the GenecCards database, 560 genes from the OMIM database and 53 genes from the TTD database were identified. All collected genes were combined and deduplicated, and 3659 target genes were predicted (Table S2). Subsequently, 75 potential DHM targets for depression were predicted in the Venny database (http://bioinfogp.cnb.csis.es/tools/venny/index.html, accessed on 18 August 2022) (Figure 3, Table S3).

### 3.3. PPI Network of DHM Targets in Depression

**Figure 4:**
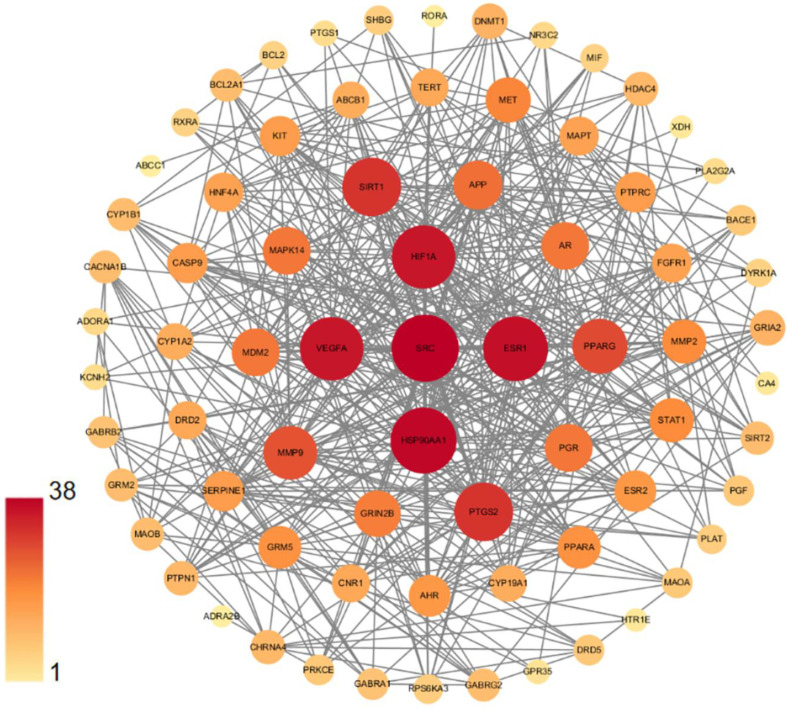
Seventy-three intersection targets predicted by Protein–protein interaction (PPI) network analysis. The size and color of the nodes indicate the size of the node degree value. The larger the node and the deeper the color red, the higher the degree value of the node.

We constructed a PPI network by importing 75 potential targets into the STRING database; the network was visualized using Cytoscape software Version 3.9.1. Figure 4 shows the 73 nodes and their interactions. Two targets, CMP-N-acetylneuraminate-beta-1,4-galactoside alpha-2,3-sialyltransferase (ST3GAL3) and galanin receptor type 3 (GALR3) are not presented in Figure 4, due to the fact that they were not connected to this network. In the network, the node represents the relevant gene, with the size and color of the node representing the value of the free degree. Proteins with a “degree” value higher than 31 were selected as the significant targets, which included proto-oncogene tyrosine-protein kinase (SRC), heat shock protein 90 alpha family class A member 1 (HSP90AA1), estrogen receptor 1 (ESR1), hypoxia inducible factor 1 subunit alpha (HIF1A), vascular endothelial growth factor A (VEGFA), silent information regulator 1 (Sirt1) and prostaglandin endoperoxide synthase 2 (PTGS2), all of which may play an important role in DHM’s action against depression.

### 3.4. GO and KEGG Pathway Enrichment Analyses for DHM-Targeted Gene Function and Signaling Pathways

**Figure 5:**
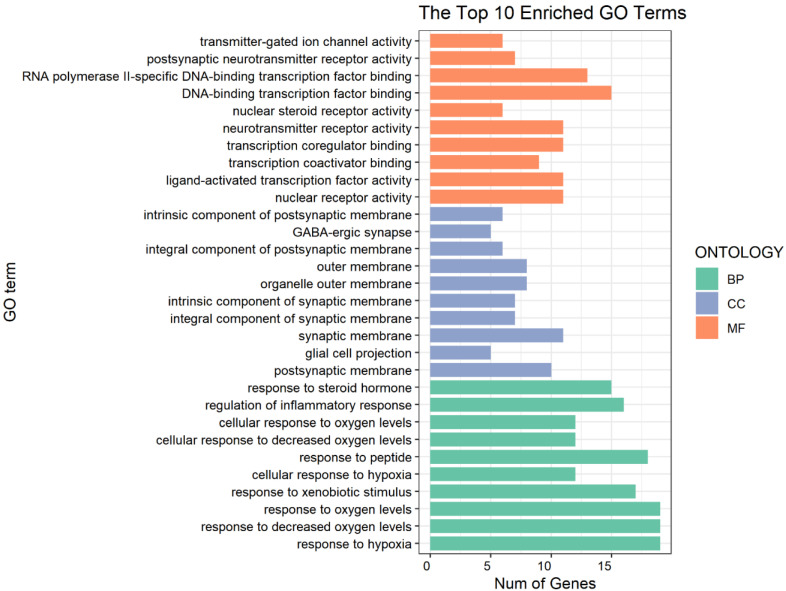
The top 10 enriched terms as shown in the GO enrichment. The *x*-axis represents the number of enriched genes in each term; the *y*-axis represents enriched terms in each category. BP: biological process; CC: cellular component; and MF: Molecular function.

**Figure 6:**
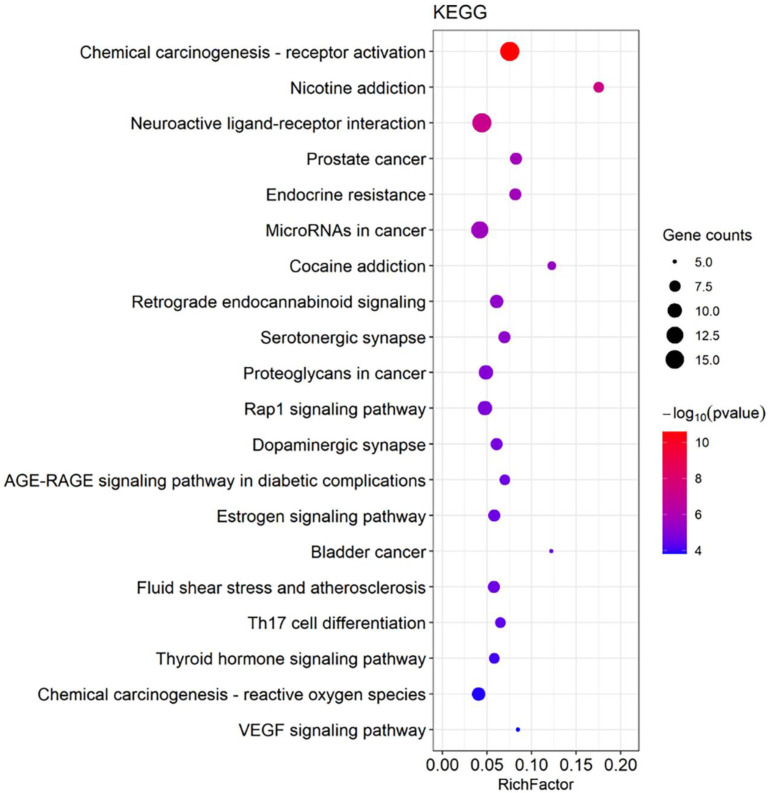
The top 20 dihydromyricetin (DHM)-mediated signaling pathways predicted by the Kyoto Encyclopedia of Genes and Genomes (KEGG) enrichment analysis.

Related biological processes and signaling pathways of DHM targets were identified using GO enrichment and KEGG pathway enrichment analyses. A total of 1503 GO terms, including 1316 of biological process (BP), 65 of cellular component (CC), and 122 of molecular function (MF), were acquired. The top 10 terms of BP, CC, and MF are shown in Figure 5. The data demonstrate that these potential targets are predominantly associated with the activities of neurotransmitter receptors, transmitter-gated ion channel and nuclear (steroid) receptors, which mediate depression [9,10,11,12,13,14]; these target genes are also possibly involved in the binding of transcription factors and transcription coactivators, which may mediate the development of depression [15]. These potential targets function mainly in the (post)synaptic membrane, GABA-ergic membrane and (organelle) outer membrane, where receptor-mediated signal transductions may regulate the development of depression [16,17]. The targets possibly play a critical role in response to steroid hormone levels, regulation of inflammation and response to oxygen levels, which are known to be involved in the development of depression [18,19,20]. Additionally, the KEGG pathway enrichment analysis was performed to uncover DHM-mediated functional mechanisms. Among the mapped top 20 signaling pathways (Figure 6), the retrograde endocannabinoid signaling, Rap1 signaling, AGE-RAGE signaling, Estrogen signaling, oxygen reactive species signaling and VEGF signaling pathways are particularly associated with the development and progression of depression [21,22,23,24,25,26].

### 3.5. Inhibition of AGE-RAGE Signaling Mediated Inflammation in CORT-Exposed Mouse Hippocampus

**Figure 7:**
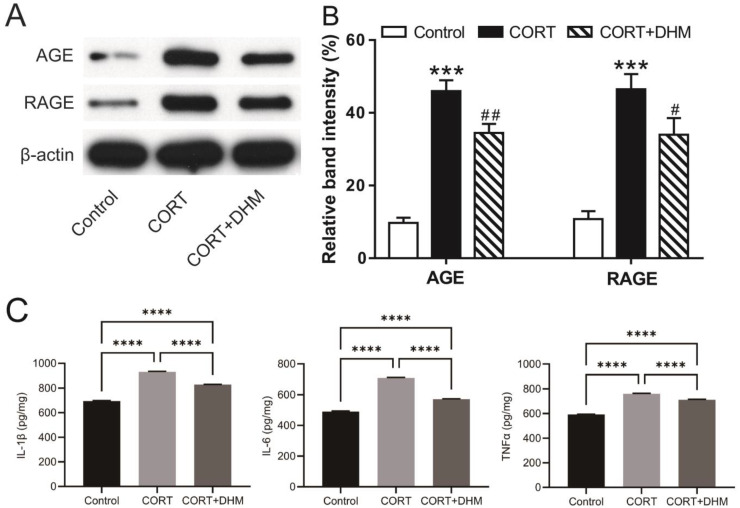
Inhibition of AGE-RAGE-mediated inflammation by DHM in the hippocampus of CORT-treated mice. (**A**) Representative immunoblots of hippocampal AGE, RAGE. (**B**) Quantification of AGE, RAGE by normalizing to β-actin. Data are expressed as mean ± SEM (n = 4 mice/group). *** *p* < 0.001, vs. the control group; ^#^ *p* < 0.05, ^##^ *p* < 0.01, vs. the CORT group. (**C**) Levels of proinflammatory cytokines, IL-1β, IL-6 and TNFα detected by ELISA. Data are expressed as mean ± SEM (n = 5 mice/group). **** *p* < 0.0001. CORT, corticosterone; DHM, dihydromyricetin.

We next examined one of the mapped top signaling pathways, AGE-RAGE, in the hippocampus of mice chronically treated with CORT. We found that the levels of AGE and RAGE proteins were significantly increased in the CORT-treated animals, compared to the control animals; DHM-treated animals had significantly lower levels of AGE and RAGE proteins compared to animals treated with CORT alone (Figure 7A,B and Figure S1). Activation of the AGE-RAGE signaling pathway can lead to upregulation of proinflammatory cytokines [27]. Consequently, we examined the production of IL-1β, IL-6 and TNFα in the hippocampus of these animals by ELISA. The levels of these proinflammatory cytokines were markedly increased in CORT-treated mice compared to the control animals; mice co-treated with DHM had a marked decrease in the expression of these cytokines (Figure 7C) compared to those treated with CORT alone.

## 4. Discussion

In the current study, we demonstrated that DHM relieved chronic CORT-induced depression-like behaviors in mice, supporting the notion that DHM has therapeutic potential for patients with depressive disorders. Additionally, network pharmacology analyses predicted that DHM’s efficacy against depression is possibly mediated by different targets and signaling pathways, of which the AGE-RAGE was further examined. We confirmed that the AGE-RAGE signaling pathway was activated and expression of proinflammatory cytokines was upregulated in the hippocampus of mice exposed to CORT, and that co-treatment with DHM counteracted these changes.

Earlier studies have demonstrated that DHM alleviates depression-like behaviors in the lipopolysaccharides (LPS)-induced acute depression mouse model and in the chronic unpredictable mild stress-induced depression model [28,29]. DHM has also demonstrated antidepression effects in a diabetic neuropathic pain and depression comorbidity model [7,30]. Consistently, our data showed that chronic CORT injection resulted in increased immobility time in the FST, while DHM administration exerted an anti-immobility effect. Considering the effect of the level of locomotor activity on immobility time in the FST, the distance traveled by the mice was measured to evaluate locomotor activity in the OFT. Our data show that all the groups traveled an equivalent distance, indicating that neither CORT nor DHM administration influenced the level of locomotor activity. These data further strengthen the anti-despair role of DHM. Additionally, our data also show that chronic CORT injection resulted in decreased sucrose preference behavior in SPT, while DHM treatment boosted sucrose preference in chronic CORT-exposed mice, suggesting an anti-anhedonia role of DHM.

Network pharmacology is a system biology approach that is used to identify potential drug candidates and drug targets and to predict their functional mechanisms [31]. Recently, it has been widely applied in the discovery of antidepressants from natural products including medicinal plants and traditional Chinese medicine [32,33]. Network pharmacology analyses showed that DHM had multiple targets associated with its anti-depression function. Among these core targets, SIRT1, HIF1A, ESR1, VEGFA, HSP90AA1 and PTGS2 are well documented to be associated with depression [26,28,34,35,36,37]. Furthermore, the KEGG enrichment approach mapped DHM-mediated signaling pathways which are possibly involved in the pathogenesis of depression. One of these pathways, AGE-RAGE, has also been suggested by the network pharmacology approach to be involved in the efficacy of herb medicines against chronic pain-related depression [32,33]. Previous studies have also demonstrated that the alleviation of depressive-like behaviors in rodent models by natural products (p-coumaric acid and melatonin) is mediated by the AGE-RAGE signaling pathway [9,38].

Inflammation plays a critical role in the development and progression of depression [19]. DHM has demonstrated an anti-inflammation capacity in various types of diseases by inactivating the NLRP-3 and NF-κB pathways and decreasing the production of proinflammatory cytokines such as IL-1β, IL-6 and TNFα [39]. Wei et al. reported that DHM reversed depressive-like behavior in LPS-treated rodents by blocking the TLR4/Akt/HIF1a/NLRP3 pathway, inactivating the NF-κB pathway and decreasing the expression of proinflammatory factors [28]. In the chronic unpredicted mild stress-induced depression mouse model, DHM’s action against depression is associated with the upregulation of brain-derived neurotrophic factor (BDNF) and inhibition of inflammation in the hippocampus [29]. Similarly, DHM treatment also upregulates BDNF expression and decreases the expression of proinflammatory cytokines in the hippocampus of rats with depression and diabetic neuropathic pain [30]. The AGE-RAGE signaling pathway also regulates inflammation via downstream activation of the NF-κB pathway and the promotion of proinflammatory cytokine generation [27]. In the current study, we found that DHM treatment decreased the levels of AGE, RAGE and proinflammatory factors in CORT-treated mouse hippocampus, suggesting that the AGE-RAGE signaling pathway was inactivated. Previous studies have reported that BDNF inhibits hippocampus inflammation in type 1 diabetic mice via the inactivation of the RAGE–NF-κB pathway [40]. Since DHM has been shown to enhance BDNF expression in two other depression rodent models [29,30], it is reasonable to conclude that DHM also stimulates BDNF expression, controls the AGE-RAGE–NF-κB axis, and inhibits inflammation in the hippocampus of CORT-exposed mice, which is worthy of further investigation.

Depression is also a common clinical feature presented in neurodegenerative disorders, including Alzheimer’s disease (AD) and Parkinson’s disease (PD) [41,42]. The depressive phenotypes in AD or PD patients are similar to that of depressive patients without AD or PD. Both AD and major depression have overlapping disease mechanisms [43,44]. DHM may therefore offer benefits to patients with neurodegenerative diseases.

In conclusion, our present study showed that DHM eased depression-like behaviors in chronic CORT-treated animals. The protection of DHM against depression may be associated with multiple targets and signaling pathways, among which the AGE-RAGE pathway possibly plays a central role. The data suggest that DHM has therapeutic potential for treating patients with depression.

## Data Availability

Not applicable.

## Supplementary Materials

The following supporting information can be downloaded at: https://www.mdpi.com/article/10.3390/cells11233730/s1; Figure S1: the image of the original Western blot for Figure 7A; Table S1: Targets of dihydromyricetin; Table S2: Targets of depression; Table S3: Intersecting targets.
