# Supplementary material for: Dihydromyricetin Attenuates Depressive-like Behaviors in Mice by Inhibiting the AGE-RAGE Signaling Pathway
Source: Cells. 2022 Nov 22;11(23):3730. doi: 10.3390/cells11233730 (PMC9738449; doi:10.3390/cells11233730)
Supplement: Supplementary file 1 [file cells-11-03730-s001.zip › cells-1999597-supplementary.pdf]

Supplementary data

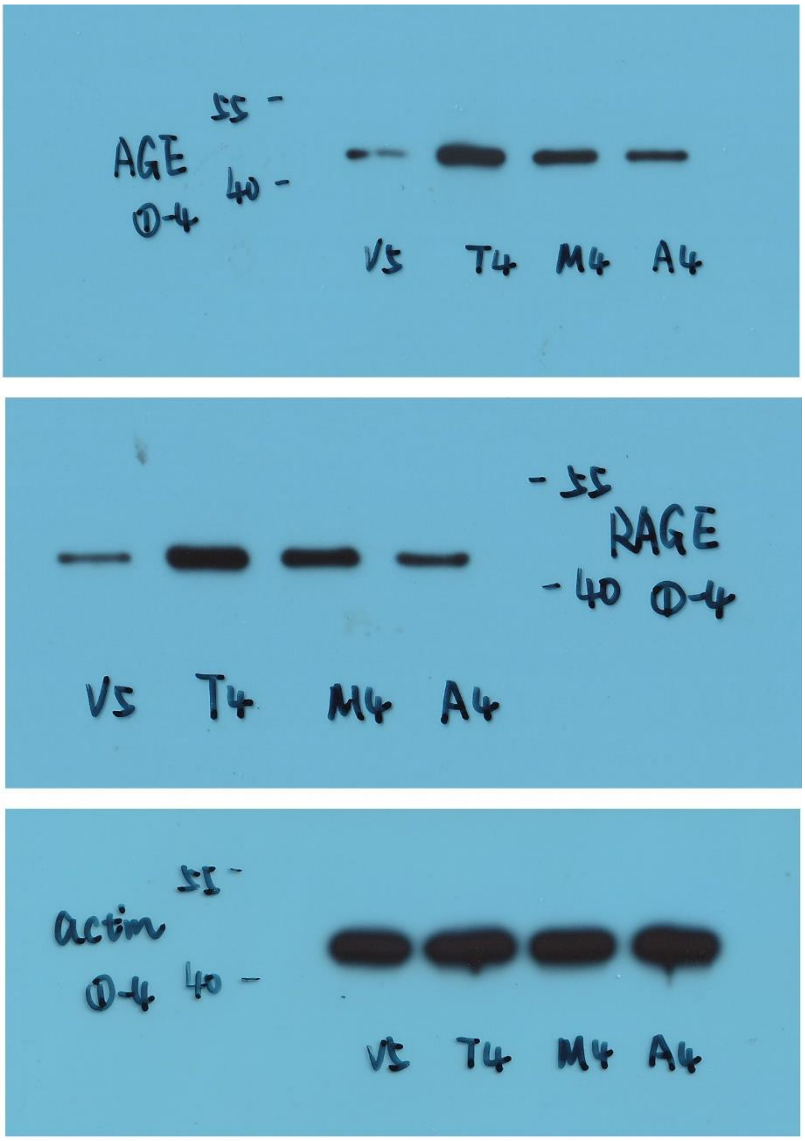

V5: Control
T4: CORT
M4: CORT+DHM
A4: a sample for another project.

Figure S1. The original Western blot images for Figure 7A.
